# Psychoacoustic classification of persistent tinnitus^☆^

**DOI:** 10.1016/j.bjorl.2017.07.005

**Published:** 2017-08-01

**Authors:** Flavia Alencar de Barros Suzuki, Fabio Akira Suzuki, Ektor Tsuneo Onishi, Norma Oliveira Penido

**Affiliations:** Universidade Federal de São Paulo (UNIFESP), Escola Paulista de Medicina (EPM), Departamento de Otorrinolaringologia e Cirurgia Cabeça e Pescoço, São Paulo, SP, Brazil

**Keywords:** Tinnitus/classification, Audiometry, Psychoacoustic/characteristics, Zumbido/classificação, Audiometria, Psicoacústicas/características

## Abstract

**Introduction:**

Tinnitus is a difficult to treat symptom, with different responses in patients. It is classified in different ways, according to its origin and associated diseases.

**Objective:**

to propose a single and measurable classification of persistent tinnitus, through its perception as sounds of nature or of daily life and its comparison with pure tone or noise, of high or low pitch, presented to the patient by audiometer sound.

**Methods:**

A total of 110 adult patients, of both genders, treated at the Tinnitus Outpatient Clinic, were enrolled according to the inclusion and exclusion criteria. Otorhinolaryngologic and Audiological, Pitch Matching and Loudness, Visual Analog Scale, Tinnitus Handicap Inventory and Minimum Masking Level assessments were performed.

**Results:**

In these 110 patients, 181 tinnitus complaints were identified accordingly to type and ear, with 93 (51%) Pure Tone, and 88 (49%) Noise type; 19 at low and 162 at high frequency; with a mean in the Pure Tone of 5.47 in the Visual Analog Scale and 12.31 decibel in the Loudness and a mean in the Noise of 6.66 and 10.51 decibel. For Tinnitus Handicap Inventory and Minimum Masking Level, the 110 patients were separated into three groups with tinnitus, Pure Tone, Noise and multiple. Tinnitus Handicap Inventory higher in the group with multiple tinnitus, of 61.38. Masking noises such as White Noise and Narrow Band were used for the Minimum Masking Level at the frequencies of 500 and 6000 Hz. There was a similarity between the Pure Tone and Multiple groups. In the Noise group, different responses were found when Narrow Band was used at low frequency.

**Conclusion:**

Classifying persistent tinnitus as pure tone or noise, present in high or low frequency and establishing its different characteristics allow us to know its peculiarities and the effects of this symptom in patients’ lives.

## Resumo

**Introdução:**

O zumbido é um sintoma de difícil tratamento, com respostas diferentes nos pacientes. É classificado de formas diversas, de acordo com a origem ou doenças associadas.

**Objetivo:**

Propor uma classificação única e mensurável do zumbido persistente, por meio da sua percepção como sons da natureza ou da vida cotidiana e da sua comparação com o tom puro ou o ruído, de *pitch* alto ou baixo, apresentados ao paciente pelos sons do audiômetro.

**Método:**

Participaram 110 pacientes adultos, de ambos os sexos, atendidos no Ambulatório de Zumbido, tendo sido observados os critérios de inclusão e exclusão. Realizada avaliação otorrinolaringológica, audiológica, *Pitch Matching* e *Loudness, Visual Analog Scale*, *Tinnitus Handicap Inventory* e *Minimum Masking Level*.

**Resultados:**

Nesses 110 pacientes foram identificadas 181 queixas de zumbido separadas por tipo e orelha, sendo 93 (51%) tipo tom puro e 88 (49%) tipo ruído; 19 de baixa frequência e 162 de alta frequência; com média do *Visual Analog Scale* no tom puro de 5,47 e ruído de 6,66; média do *Loudness* do tom puro de 12,31 dBNS e ruído de 10,51 dBNS. Para o *Tinnitus Handicap Inventory* e o *Minimum Masking Level* os 110 pacientes foram separados em três grupos com zumbido, tom puro, ruído e múltiplo, com a média do *Tinnitus Handicap Inventory* maior no grupo com zumbido múltiplo com 61,38. Para o *Minimum Masking Level* foram usados os ruídos mascaradores tipo *White Noise* e *Narrow Band* nas frequências de 500 Hz e 6000 Hz. Houve semelhança entre os grupos com tom puro e múltiplo. No grupo de ruído foram encontradas respostas diferentes quando usado o *Narrow Band* em frequência baixa.

**Conclusão:**

Classificar o zumbido persistente em tom puro ou ruído, presentes em frequência alta ou baixa e estabelecer suas diferentes características nos permitem conhecer suas particularidades e a repercussão desse sintoma na vida dos pacientes.

## Introduction

The increase in the worldwide population and life expectancy challenges our existence and may cause a crisis in the public and private health systems in both, poor and rich countries. Researchers around the world are looking for solutions to improve the population's accessibility to the health system, using standardized, simplified and easy-to-use classifications and treatments.

Regarding tinnitus, the first step in this investigation is to find a cause and effect relationship, seeking the etiological treatment of the underlying disease to be able to suppress or inhibit tinnitus. When it persists, the patient often returns home without any assistance or is referred to some psychological treatment to learn how to live with this sensation.

Although the current approach to tinnitus is based on symptomatic approaches, its classification continues to be established by its origin or etiology.^1^, ^2^, ^3^ In the Clinical Practice Guideline Tinnitus *–* CPGT, Tunkel et al. (2014)^4^ addressed the importance of classification to direct the treatment, considering individuals amenable to treatment those with persistent and uncomfortable tinnitus for more than 6 months. However, they have not shown scientific evidence of how to manage patients with sequel or idiopathic tinnitus. In these cases, the treatment guidance would have to be based only on the symptom, defined by the patients as the perception of a sound when there is no sound source present.^1^, ^5^, ^6^

For this classification, the perception of tinnitus is observed as being similar to a whistle, a noise or other sound with characteristics similar to certain specific sound frequencies. Some studies have shown that these distinct characteristics of tinnitus perception have different reactions in the central nervous system (CNS). Vanneste et al., using electroencephalography (EEG), mapped tinnitus through the electrical brain responses observed at the generation of different types of tinnitus, and found distinct responses to tinnitus similar to pure tone and noise,^7^ which corroborates the importance of separating these symptoms.

To determine the loudness and correlate it with the degree of discomfort, to assess the impact it has on some aspects of the patient's life, and the possibility of masking them are crucial to understand the tinnitus symptom. The use of tinnitus quantification questionnaires and how it affects some aspects of patients lives, such as the Visual Analogs Scales – VAS,^8^ the Tinnitus Handicap Inventory – THI^9^, ^10^, ^11^ and psychoacoustic measures such as Pitch Matching, Loudness and Minimum Masking Level – MML, are of enormous value, but because they use different measurement methods, demand time, training of the examiner and specific expensive equipment,^12^ their therapeutic indication becomes imprecise and random. It is therefore important that more research is performed on how to assess, perceive and classify tinnitus.

The objective of the present study was to propose a unique and measurable classification of persistent tinnitus, through its perception as sounds of nature or daily life and its comparison with pure tone or noise, of high or low pitch, presented to the patient by audiometer sounds.

## Methods

This was a cross-sectional study, with adult patients of both genders from the Tinnitus Outpatient Clinic, from July 2011 to September 2015, and approved by the Research Ethics Committee of the institution under protocol n. CEP 1333/10. Patients were instructed on all the research procedures and signed the free and informed consent form.

Inclusion criteria were: adult patients of both genders, ranging in age from 18 to 80 years, with persistent and continuous tinnitus for more than 6 months and without hearing loss or with mild to moderate sensorineural hearing loss and asymmetry <40 decibel hearing level (dBHL) among all frequencies to avoid possible technical bias when using masking; no alterations in the external and/or middle ear and no decompensated diseases associated with tinnitus. The noise masking, Narrow Band noise, was the same as that used by the patient to classify the tinnitus as Noise.

An Interacoustics Audiometer – Model AC40 and an Interacoustics Immittance Audiometer – model AZ7 were used, both of which are calibrated annually.

Patients were submitted to anamnesis, otorhinolaryngology evaluation, tonal and vocal audiometry, immittance tests with stapedial muscle reflexes, Pitch and Loudness Matching, Visual Analog Scale (VAS), Minimum Masking Level (MML) and Tinnitus Handicap Inventory (THI). They also answered a sociodemographic and clinical questionnaire that contained data on tinnitus: whether the symptom onset was sudden or gradual, whether its perception was of a low or high frequency sound, which side was affected and what its resemblance to daily sounds was.

Based on the comparative answers acquired through the psychoacoustic measures obtained with the audiometer, the tinnitus characteristics were established: the pitch, if similar to Pure Tone (PT) or Noise, with a high or low frequency, and the Loudness, measured with attenuations of 5 dBSL and recorded in Decibel Sensation Level (dBSL). The level of annoyance caused by the tinnitus was assessed by the Visual Analog Scale (VAS).

For these measurements, the responses were analyzed in each ear, ipsilaterally and by type of tinnitus, with some patients presenting up to four responses, being similar to PT and Noise, in the right and left ears. The results were obtained by counting each response, so that the number of responses was higher than the number of patients. These responses were correlated to the responses regarding the type of tinnitus reported by the patient in the questionnaire and the psychoacoustic characteristics obtained through the audiometer.

Subsequently, these patients were divided according to the type of tinnitus into three groups: Pure Tone Group (PTG), Noise (NG) and Multiple Tinnitus, Pure Tone and Noise Group (PTNG). Each patient belonged to a single group, with the number of total responses equal to the number of patients. After being separated by gender, age and side of the tinnitus complaint, the THI and MML procedures were performed, and their characteristics were studied within each group and between groups. Regarding the THI, in order to obtain a better statistical evaluation, it was measured in values from 0 to 100, instead of degrees from 1 to 5.

To assess the MML, a numerical variable that evaluates the patient's CNS ability to suppress or mask tinnitus, three types of masking noises were used: White Noise (WN), Low Frequency Narrow Band (LFNB) at the frequency of 500 Hz and the High Frequency Narrow Band (HFNB) at the frequency of 6000 Hz. The test was always simultaneous in both ears and started with the same intensity in dBSL, with attenuations of 5 dBSL.

In the statistical analysis, the chi-square test was used to evaluate the distribution by gender, side of tinnitus and the association of Pure Tone and Noise tinnitus with the high and low frequencies. The non-parametric Mann–Whitney test was performed to evaluate the VAS and Loudness measurements. The non-parametric Kruskal–Wallis tests were used for the mean age and THI, and Tukey's multiple comparisons were used in THI to detect differences between means. The non-parametric Friedman test was used for MML measurements, with Tukey's multiple comparisons as a complement, when significant.

## Results

A sample of 110 patients with continuous and persistent chronic tinnitus was analyzed. Of these, 66 (60%) were females and 44 (40%) were males. These 110 patients showed 181 responses of the tinnitus complaint, in which the presence of tinnitus type PT was observed in 93 (51%) of the responses, and Noise in 88 (49%), including all responses and considering the presence of tinnitus on the right side and on the left side.

In relation to low pitch sensation, 19 responses were obtained at the frequencies of 250 Hz, 500 Hz or 1000 Hz corresponding to the sound reported as bass in the sociodemographic and clinical questionnaire. With the high pitch sensation, 162 responses were found that correspond to frequencies of 2000 Hz, 3000 Hz, 4000 Hz, 6000 Hz, 8000 Hz, 10,000 Hz or to WN type, compared to the high frequency sound (Table 1).

**Table 1:** Correlation of Pure Tone or Noise tinnitus with their characteristics and with low and high frequencies.

Subjective characteristics	Pitch	High frequency		Low frequency		Chi-square test (*p*)^a^	Total	
		*n*	%	*n*	%		*n*	%
Ship horn	250 Hz/500 Hz	0	0%	4	100%	*p* < 0.001	4	4.3%
Whistle/cicada/insect	2 kHz/3 kHz/4 kHz/6 kHz/8 kHz/10 kHz	89	100%	0	0%		89	95.7%
Car engine/wave/airplane/waterfall	250 Hz/500 Hz/1 kHz	0	0%	15	100%		15	17%
Bee/wheezing/pressure cooker/rain	2 kHz/3 kHz/4 kHz/6 kHz/8 kHz	73	100%	0	0%		73	83%
*n*		162	100%	19	100%		181	100%

^a^ Chi-square test. *p* < 0.001. Significance 0.05%.

*n*, number of responses to tinnitus similar to Pure Tone (PT) or Noise.

There were 93 responses of sounds similar to PT, 4 (4.3%) of Low Pitch and 89 (95.7%) of High Pitch. All patients who reported tinnitus perception as the characteristic of “ship horn” were associated with PT at the low frequencies of the audiometer and all those who perceived it as “whistle/cicada/insect”-like sounds had their responses associated with PT at the high frequencies.

There were 88 responses of tinnitus similar to Noise, 15 (17%) at low frequency and 73 (83%) at high frequency. All perceptions with the characteristic of “car engine/airplane/wave/waterfall” were associated with the Noise sound at the low frequencies of the audiometer and all those with the characteristics of “bee/wheezing/pressure cooker/rain” were associated with the sound of noise at high frequencies.

When the type of tinnitus was associated to the degree of annoyance measured through the VAS, the mean for tinnitus similar to PT was 5.47 and, for tinnitus similar to Noise, of 6.66 (Table 2). This response was significantly higher for tinnitus perceived as Noise than for the one perceived as PT.

**Table 2:** Comparison of the 181 responses of tinnitus perception as Pure Tone or Noise in relation to the Visual Analog Scale (VAS).

VAS	Pure Tone	Noise	Mann–Whitney test (*p*)	Result
Mean (0–10)	5.47	6.66	*p* = 0.002^a^	PT < Noise
Standard-deviation	2.47	2.35		
*n*	93	88		Significant

a Mann–Whitney test. Significance: 0.05%. *n*, number of responses to tinnitus similar to Pure Tone (PT) or Noise.

When analyzing tinnitus intensity in the 181 responses obtained in this study, we found the mean Loudness for tinnitus similar to PT of 12.31 dBSL and for the one similar to Noise of 10.51 dBSL (Table 3). It was verified that the values of this intensity were significantly higher for tinnitus perceived as pure tone.

**Table 3:** Comparison of 181 responses of tinnitus perception as Pure Tone or Noise in relation to Loudness.

Loudness	Pure Tone	Noise	Mann–Whitney test (*p*)	Result
Mean (dBSL)	12.31	10.51	*p* = 0.016^a^	PT > Noise
Standard-deviation	5.34	5.14		
*n*	93	88		Significant

a Mann–Whitney test. Significance: 0.05%. *n*, number of responses to tinnitus similar to Pure Tone (PT) or Noise.

These 110 patients were separated into groups, according to the type of tinnitus present. In the PTG, of the 45 (41%) patients, 60% were females and 40% males, with a mean age of 54.3 years. Of these, 25 (55.4%) patients had bilateral tinnitus. In the NG, of the 49 patients (45%) found, 57% were females and 43% were males, with a mean age of 53.4 years, with a higher incidence of tinnitus on the left side, with 22 (45%) patients. In the PTNG, of the 16 (14%) patients, 69% were females and 31% were males, with a mean age of 52.4 years. There was a higher incidence of tinnitus on the left side, with 7 (43.8%) patients and bilateral tinnitus, with 8 (50%) patients.

The impact of tinnitus on some life aspects of the 110 patients evaluated by THI was greater in the PTNG with multiple tinnitus (pure tone + noise), with a THI mean of 61.38. In the PTG, the mean was 37.42 and in the NG, it was 46.04 (Table 4). When analyzing the results of the multiple comparisons, no significant difference was found between the PTG and NG, or between the NG and PTNG, but there was a statistically significant difference between the PTG and the PTNG.

**Table 4:** Comparison between the Tinnitus Handicap Inventory of three groups, Pure Tone (PTG), Noise (NG) and Pure Tone + Noise (PTNG).

	PTG	NG	PTNG	Kruskal–Wallis test (*p*)	Tukey's multiple comparisons (*p*)	Result
Mean (0–100)	37.42	46.04	61.38	*p* = 0.009^a^	PTG × NG. *p* = 0.229	
Standard-deviation	21.56	28.81	23.26		PTG × PTNG. *p* = 0.004^a^	PTG = NG < PTNG
*n*	45	49	16		NG × PTNG, *p* = 0.093	

a Kruskal–Wallis test and Tukey's multiple comparisons. Significance of 0.05%. *n*, number of patients with tinnitus in all three groups.

The three groups were similar in relation to the intensity required to mask tinnitus when WN and HFNB masking noise were used. In relation to the low frequency masking noise, LFNB, the NG required smaller values than the PTG and PTNG groups, with MML LFNB in PTG = 30.4 dBSL; in the NG = 23.8 dBSL and in PTNG = 31.6 dBSL (Table 5). The association of the three types of masking noise, WN, LFNB and HFNB, was evaluated with the high pitch and low pitch tinnitus within each group alone.

**Table 5:** Comparison of three groups, Pure Tone (PTG), Noise (NG) and Pure Tone + Noise (PTNG), in relation to the intensity used by the Minimum Masking Level with White Noise (MML WN), Narrow Band Low Frequencies (MML LFNB) and Narrow Band High Frequencies (MML HFNB).

	Mean (dBSL)	GPT	GN	GPTN	Kruskal–Wallis test (*p*)	Tukey multiple comparisons (*p*)	Result
MML WN	Mean	14.9	14.8	17.5			PTG = NG = PTNG
	Standard deviation	8.8	9.8	8.2	0.300		
	n	45	49	16			

MML LFNBL	Mean	30.4	23.8	31.6		PTG × NG. *p* = 0.022^a^	PTG = PTNG > NG
	Standard deviation	12.7	11.2	12.2	0.004^a^	PTG × PTNG. *p* = 0.945	
						NG × PTNG. *p* = 0.066	
	n	45	49	16			

MML HFNB	Mean	14.2	16.6	19.1			PTG = NG = PTNG
	Standard deviation	8.6	13.7	10.2	0.255		
	n	45	49	16			

a Kruskal–Wallis test and Tukey's multiple comparisons. Significance of 0.05%. *n*, number of patients with tinnitus in all three groups.

In patients with high-frequency tinnitus, 42 patients were from the PTG group, 39 from the NG and 14 patients from the PTNG, with the characteristics of high frequency for PT and Noise (Table 6). These patients showed the same association found in the groups as a whole, with the mean MML intensity using the LFNB masking noise, being higher than the WN and HFNB mean (Table 6).

**Table 6:** Comparison of the Minimum Masking Level (MML) intensity using White Noise (MML WN), Narrow Band low frequencies (MML LFNB) and Narrow Band high frequencies (MML HFNB) in relation to tinnitus perception at the high frequencies in three groups, Pure Tone (PTG), Noise (NG) and Pure Tone + Noise (PTNG).

	Mean (dBSL)	MML WN	MML LFNB	MML HFNB	Friedman's test (*p*)	Tukey multiple comparisons (*p*)	Result
High-frequency PTG	Mean	15,24	31,07	14,64		WN × LFNB *p* < 0.001^a^	
	Standard deviation	8,900	12,761	8,582	<0.001^a^	WN × HFNB *p* = 0.877	WN = HFNB < LFNB
						HFNB × LFNB *p* < 0.001^a^	
	*n*	42	42	42			

High-frequency NG	Mean	13,08	24,23	12,05		WN × LFNB *p* < 0.001^a^	
	Standard deviation	7,832	10,671	8,006	<0,001^a^	WN × HFNB *p* = 0.485	WN = HFNB < LFNB
						HFNB × LFNB *p* < 0.001^a^	
	*N*	39	39	39			

High-frequency PTNG (pure tone and noise)	Mean	15,00	29,29	16,79		WN × LFNB *p* < 0.001^a^	
	Standard deviation	4,804	11,242	8,684	<0,001^a^	WN × HFNB *p* = 0.718	WN = HFNB < LFNB
						HFNB × LFNB *p* < 0.001^a^	
	*N*	14	14	14			

a Friedman's test and Tukey's multiple comparisons. Significance of 0.05%. *n*, number of patients with tinnitus in all three groups.

Regarding patients with low-frequency tinnitus, three patients were found in the PTG group, 10 patients in the NG and two patients in the PTNG, with distinct characteristics of high frequency pure tone tinnitus and low frequency Noise tinnitus (Table 7). Patients with low-frequency tinnitus from the PTG and PTNG groups had the same intensity association with masking noises, WN = HFNB < LFNB, but statistical analysis could not be performed because of the low incidence. However, it was observed that those who had multiple PT tinnitus with high frequency sensation associated with low frequency Noise tinnitus had greater difficulty in masking, requiring a high noise masking intensity (MML). Patients with low-frequency tinnitus required more intensity to mask it with HFNB and WN than those with high-frequency tinnitus.

**Table 7:** Comparison of the Minimum Masking Level (MML) intensity using White Noise (MML WN), Narrow Band low frequencies (MML LFNB) and Narrow Band high frequencies (MML HFNB) in relation to the perception of tinnitus at the low frequencies in the Pure Tone (PTG) and Noise (NG). In the multiple group (PTNG), the tinnitus at the high frequencies for Pure Tone and low frequencies for Noise.

	Mean (dBSL)	MML WN	MML LFNB	MML HFNB	Friedman's test (*p*)	Tukey's multiple comparisons (*p*)	Result
Low-frequency PTG	Mean	10.00	21.67	8.33	–	–	–
	Standard deviation	5.000	7.638	7.638	–	–	–
	*n*	3	3	3			

Low-frequency NG	Mean	21.50	22.00	34.50		WN × LFNB. *p* = 0.984	
	Standard deviation	13.754	13.581	16.907	0.005^a^	WN × HFNB. *p* < 0.001^a^	WN*p* = LFNB < HFNB
						HFNB × LFNB. *p* = 0.001^a^	
	*n*	10	10	10			

Low-frequency (noise) + High-frequency (pure tone) PTNG	Mean	35.00	47.50	35.00	–	–	–
	Standard deviation	0.000	3.536	0.000	–	–	–
	n	2	2	2			

a Friedman's test and Tukey's multiple comparisons. Significance of 0.05%. *n*, number of patients with low frequency tinnitus in PTG and Noise and high frequency for PT and low frequency for Noise in the PTNG.

## Discussion

The absence of a specific classification to score the assessment criteria for tinnitus characteristics, associated with the lack of measurement parameters, makes it impossible to perform a comparative analysis of improvement or worsening of tinnitus with the several available therapies. Treatments currently reported in the literature, such as acupuncture, transcranial stimulation and drug therapies^4^, ^13^, ^14^, ^15^ do not have a consensus, showing benefits only in selected cases. This is probably due to the lack of standardization for the selection of different types of tinnitus, thus being necessary to adopt a new classification criterion to better evaluate and target these treatments.

Stouffer and Tyler (1990) and Shulman (1997) found several subjective descriptions, difficult to be measured and compared.^2^, ^16^ This study was able to show significance for all tinnitus characteristics with the sound produced by the audiometer, identified as PT or Noise, with low or high frequency sensation. Unlike Vernon and Meikle (2003), who found greater presence of low-frequency tinnitus in the Noise type,^17^ this study found a higher presence of tinnitus responses similar to the high frequency sensation, both for PT and for Noise (Table 1).

For many years tinnitus was believed to be related only to changes in the auditory nerve and cochlea. The use of imaging and EEG exams has demonstrated the possible involvement of central mechanisms in tinnitus generation and perception, with different brain activities for different types of tinnitus.^7^, ^18^ This has corroborated the importance of evaluating and studying the distinct characteristics of PT and Noise tinnitus.

When analyzing the VAS in relation to the type of tinnitus, it was observed that the most uncomfortable resembles a tinnitus similar to Noise (Table 2), the same findings of Vanneste et al. (2010).^7^

The Noise tinnitus, because it is a frequency spectrum, involves a larger area, with greater EEG activity^7^ and consists of aperiodic waves, of random movements that do not repeat themselves.^19^ This makes it more difficult for the CNS to get habituated. This was observed in the study by Barros Suzuki et al. (2016), demonstrating that PT tinnitus (whistle) has a better response to the treatment with sound therapy than the Noise type.^20^

When the loudness responses were analyzed, the PT mean was statistically significant higher than that of the Noise tinnitus (Table 3), which may be justified by the greater presence of bilateral tinnitus in the PTG group.

Vanneste et al. (2010) and Balkenhol et al. (2013), when studying the EEG of patients with tinnitus, observed a difference in the responses of PT and Noise tinnitus and found patterns of distinct brain activities for tinnitus intensity and discomfort,^7^, ^18^ which can also justify the fact that we have different answers, with the annoyance measured by the VAS worse in the Noise tinnitus and the intensity (loudness) worse in the PT type.

The impact of tinnitus on some aspects of patient life as measured by the THI and the masking of tinnitus through MML cannot be assessed separately by ear. Thus, to analyze these explanations of the tinnitus characteristics in the 110 assessed patients, these were separated into three groups, PTG, NG and PTNG. There were a greater number of females in these three groups. The groups are homogeneous in relation to gender and age and heterogeneous in relation to laterality, with bilateral tinnitus found more often in the PTG.

The THI was chosen because it is easily applicable and has been translated and validated in several languages, including Portuguese.^10^, ^11^, ^21^

Although studies such as the one by Figueiredo et al. (2009) correlated THI with VAS,^8^ by separating patients into groups, this association was not observed. While VAS was worse for those with Noise Tinnitus, THI did not show a significant difference between PTG and NG groups, but showed a much higher mean in the group with both types of tinnitus, PTNG (Table 4). When comparing the three groups, it is observed that the THI score is significantly higher in the PTNG group. These findings were similar to that of Lim et al. (2010).^22^

The MML analysis with three types of masking noise was performed considering the findings of Feldmann (1971) and Vernon and Meikle (2003). They concluded that many patients had tinnitus masked by external noises of frequencies similar to their tinnitus and that the use of MML would be better when used with frequencies higher than speech.^17^, ^23^

When comparing the three types of masking noise used: WN, LFNB and HFNB, in the three groups of patients, there was a homogeneity regarding the proportion of masking noise used. The MML with WN and with HFNB showed similar and lower mean values than the MML intensities with LFNB, which required more intensity to mask the tinnitus (Table 5).

However, when the amount of intensity used was analyzed, a difference was found between the groups when LFNB was used. While with WN and HFNB the three groups obtained similar MML averages, PTG = NG = PTNG, with low frequency masked noise, LFNB, the proportion was different, PTG = PTNG > NG (Table 5). In this case a lower intensity of low frequency masking noise was required to mask the tinnitus noise group (NG).

The NG was the group with the highest incidence of low pitch tinnitus, which may have contributed to lower that average, following the same premise that the masking noise that requires less intensity to suppress the tinnitus is the one that resembles the tinnitus itself.

When analyzing the MML intensity variables using masking noise in relation to high pitch tinnitus, the ratio was WN = HFNB < LFNB (Table 6). In the PTG and PTNG groups, the low incidence of patients with low pitch tinnitus did not allow the statistical analysis. However, in the NG, for low pitch tinnitus patients, the WN = LFNB < HFNB ratio was found, requiring less intensity to mask the tinnitus when performing the MML with LFNB (Table 7).

In the PTNG, with both types of tinnitus, two patients were recorded with the characteristics of PT with High Pitch and Noise with Low Pitch, whereas there were 14 patients with PT and Noise tinnitus with High Pitch. The presence of tinnitus with high pitch caused the WN = HFNB < LFNB ratio to be maintained.

The two patients with high frequency tinnitus for PT and low frequency for Noise were those who required more intensity of MML to suppress tinnitus with all types of masking noises. Although there are only two cases, and it was not possible to analyze from a statistical point of view, it may be assumed that multiple tinnitus of different characteristics is the most difficult to mask and that these patients may present greater difficulty to be treated.

All these findings confirm what Feldman (1971) and Barros Suzuki et al. (2016) had already observed. The patient needs less intensity when the noise used to mask it is the closest to the frequency of the tinnitus to be masked.^20^, ^23^ As most of them were found at the high frequencies, the MML, using low frequency masking noise, was the most difficult to suppress the tinnitus.

To know the differences between the PT and Noise types of tinnitus and to notice some differences such as the fact that the pure tone has a greater sensation of intensity and bilateral presence; Noise tinnitus has a greater degree of discomfort; multiple tinnitus affects more some aspects of the patient's life and requires less intensity to mask it when the frequency is similar to the frequency of the masking noise, is of great importance to determine the best treatment for the patients, especially when the data regarding the associated pathologies are poorly defined.

These evaluations can be performed by any professional trained on a single-channel audiometer, except for MML, which is a binaural test and requires a two-channel audiometer.

## Conclusion

To classify persistent tinnitus as pure tone or noise, present at high or low frequency and establish its different characteristics allows us to know its peculiarities and the effect of this symptom on the patients’ lives, leading us to a treatment direction.

## Conflicts of interest

The authors declare no conflicts of interest.
